# Three approaches to assessing dietary quality in Mexican adolescents from 2006 to 2018 with data from national health and nutrition surveys

**DOI:** 10.1017/S1368980024000648

**Published:** 2024-03-11

**Authors:** Elsa Berenice Gaona-Pineda, Nancy López-Olmedo, Hortensia Moreno-Macías, Teresa Shamah-Levy

**Affiliations:** 1 Center for Evaluation and Survey Research, National Institute of Public Health of Mexico, Cuernavaca, Mexico; 2 Master’s and Doctoral Program in Medical, Odontological and Health Sciences, National Autonomous University of Mexico, Mexico City, Mexico; 3 Center for Population Health Research, National Institute of Public Health of Mexico, Cuernavaca, Mexico; 4 Autonomous Metropolitan University, Iztapalapa, Mexico

**Keywords:** Dietary quality, Adolescents, Mexico, Trends

## Abstract

**Objective:**

To assess trends in the dietary quality of Mexican adolescents from 2006 to 2018, both overall and by sociodemographic indicators, using adaptations of the EAT-Lancet Planetary Health (PH) recommendations, optimal intake estimated by the Global Burden of Disease (GBD) and 2015 Mexican Dietary Guidelines (MDG) in nationally representative samples.

**Design:**

Using dietary data from a semi-quantitative FFQ, dietary quality indexes were constructed as adaptations of three dietary intake recommendations. Trends in adherence to recommendations were evaluated with multivariate quantile regression models with survey year as the main independent variable and adjusted for age, sex, energy intake, dwelling area, geographical region, household assets condition, and student/non-student status. *P* values and CI were Bonferroni-corrected.

**Setting:**

Mexico.

**Participants:**

Non-pregnant or lactating adolescents aged 12–19 years (*n* 16 520).

**Results:**

Adherence to the PH index was about 40 %, GBD was nearly 35 % and MDG was about 37 %. The lowest adherences were for added sugars, sugar-sweetened beverages, nuts and seeds, red meats, processed meats, and legumes (<28 %). No 2006–2018 trends in total adherence were found in any index. Nevertheless, negative adherence trends were identified for poultry (*β* = –2·4), and saturated fats (*β* = –0·93), and positive for unsaturated oils (*β* = 1·23), in the PH. In MDG, relevant trends were found for plain water (*β* = 1·63) and foods rich in fats (*β* = –1·24).

**Conclusions:**

Mexican adolescents have demonstrated poor dietary quality by these three approaches. Therefore, this population has a high-risk profile for diet-associated chronic diseases. Further research and appropriate public policies are needed.

Poor diet is a direct cause of overweight and obesity and a risk factor for nutritional deficiencies and many diet-associated chronic diseases, including some types of cancer^(1)^. A high-quality diet is key to maintaining adequate health and nutritional status, especially in the context of epidemiological and nutritional transitions^(2)^. Diet is critical for growth and development among adolescents, and the dietary patterns established during this stage affect health, including the potential risk of developing overweight and obesity^(3)^. In Mexico, the prevalence of overweight and obesity in adolescents was 38·8 % in 2018 and rose to 43·8 % in 2020^(4,5)^. It has been reported that adolescents with overweight or obesity have metabolic alterations, among other health effects, and that these conditions are more likely to remain and worsen in adulthood^(3)^. Therefore, it is important to assess and monitor the dietary quality of this population.

One way to evaluate dietary quality is through indexes. Those allow comparisons between the consumption of food groups and nutrients of interest against their recommended values for specific age-group populations^(6)^. Multiple dietary quality indexes have been described in the scientific literature. Some of the most commonly referenced are those derived from dietary guidelines for the USA such as the Healthy Eating Index (HEI-2015)^(7)^. Some indexes cater to specific dietary patterns, such as the Mediterranean Diet Quality Index^(8)^. Recently, the EAT-Lancet group released the Planetary Health (PH) recommendations^(9)^, which seek to promote changes in the food system to enable more healthy and sustainable diets^(9)^. Other potential consumption recommendations that may be of interest for evaluating dietary quality are the optimal intakes estimated by the Global Burden of Disease (GBD) with information across 195 countries related to mortality risk and years of life lost due to disability^(10)^.

Information on dietary quality among Mexican adolescents is limited to a few surveys or studies with small samples. Some available include Rodríguez *et al.* ^(11)^ using the HEI-2010 and Aljahdali *et al.* ^(12)^ using the Dietary Approaches to Stop Hypertension Score(DASH), alternate Mediterranean diet, and Dietary Inflammatory Index in a Mexico City birth cohort. In addition, the adolescents’ dietary quality trends over time and through sociodemographic strata have not been explored. This could be necessary to identify those at higher risk of adverse health outcomes and allow interventions focused on them.

Therefore, the objective of this study was to assess the dietary quality in nationally representative samples of Mexican adolescents from 2006 to 2018, both overall and by sociodemographic indicators, using adaptations of recommendations put forth in the PH, GBD, and 2015 Mexican Dietary Guidelines (MDG)^(13)^. MDG were used because they were the dietary guidelines recommended in Mexico at the time of our analysis, and it was important to compare them.

## Methods

### Design and population

We studied adolescents aged 12–19 years in samples from the 2006, 2012, 2016, and 2018 National Health and Nutrition Surveys (ENSANUT by its acronym in Spanish). The ENSANUT surveys have a multistage cluster and probability sampling design and are representative at the household and population levels in Mexico. In the first stage, basic geostatistical areas (primer sampling units) were selected for each dwelling stratum and region of the country. In the second stage, street blocks in urban settings or clusters of houses in rural settings were selected. In the third stage, dwellings were selected, and one person was selected from each age group of interest. Dietary data allowed for representative estimations at the national, regional and rural/urban levels. Further information regarding the ENSANUT design can be found elsewhere^(14–17)^.

This analysis included non-pregnant, non-lactating adolescents aged 12–19 years with information on dietary intake, weight and height.

### Dietary data method and cleaning

Dietary data were collected through a semi-quantitative FFQ (SFFQ), which included a list of 140 foods and beverages for the 2012–2018 surveys and 101 foods and beverages for the 2006 survey. For each food item, we asked the number of days consumed (0–7 d), times per d (1–6 times), portion size consumed in cups, tablespoons, or pieces, and the number of portions consumed. The 7-d average consumption was then estimated in grams or millilitres of each food or beverage. Subsequently, based on food nutritional composition tables^(18,19)^, we calculated energy and nutrient intakes per food item consumed and the 7-d average per d. This SFFQ was validated in previous studies and showed strong correlations with nutrient intakes derived through 24-h recall^(20)^.

Dietary data were cleaned and processed according to statistical cut-off points and values for energy and nutrient requirements, according to criteria used in ENSANUT^(21)^. Intakes of 4 sd or more above the mean consumption by sex for each food or beverage were considered implausible and excluded. Consumption above a limit, defined as the 1·5 × 99th percentile of consumption distributions, was also classified as implausible. This consumption was imputed with a random value between the 95th percentile and the 1·5 × 99th percentile limit^(21)^. Participants with seven or more foods or beverages (5 % of those listed in SFFQ) with imputed consumption values were excluded.

In addition, participants with implausible values for intakes of total energy, protein and fibre were excluded from this analysis; energy intakes of half of the BMR or above 3 sd from the mean of energy requirements were defined as implausible. For protein and fibre, intakes higher than 3 sd from their required or adequate intake by sex and age were considered implausible, according to the methodology reported by Ramírez *et al.* ^(22)^

Under these criteria, and including only participants with valid BMI according to WHO criteria^(23)^, total losses to the sample sizes were 17·4 % in 2006, 13·7 % in 2012, 11·5 % in 2016 and 5·3 % in 2018 (see online supplementary material, Supplemental Fig. 1).

### Dietary quality indexes

To construct the dietary quality indexes, foods and beverages consumed were classified into the components of each index (see online supplementary material, Supplemental Table 1). Food items from the SFFQ that included mixed dishes, such as Mexican dishes, sandwiches and hamburgers, were disaggregated into their ingredients according to averaged ingredients and amounts recipes based on 24-h recalls applied in previous ENSANUT (average recipes have not been published)^(11,24,25)^. Each ingredient was classified into the corresponding component.

Dietary quality indexes were constructed and adapted considering the context of the Mexican diet and the instrument used to collect dietary data as follows:

Scores were assigned according to four dimensions in which the components of each index were classified: healthy without waste, healthy, unhealthy in excess and unhealthy. Healthy without waste includes healthy food groups like fruits, vegetables, and nuts and seeds. The score for this dimension increases as consumption increases, but to avoid food waste as recommended by the PH, the score decreases as intake exceeds the recommended range of consumption up to a limit where a minimum score was assigned. This limit was defined as the 85th percentile between the upper range of the recommended value and the maximum intake recorded in the survey sample. This dimension was applied only to the PH index. The healthy dimension includes food groups such as high-fibre cereals, legumes, fruits and vegetables. Components of this dimension are considered healthy without penalty to the score when consumption is above the recommended value. This implies that the score increases with consumption, and a maximum score is assigned once the recommended value is reached. The unhealthy in excess includes elements for health, although it is not advisable to exceed the recommended value. Some examples of this dimension are tubers and dairy products. Once consumption exceeded the recommended value, a score of 0 was assigned. Finally, the unhealthy dimension includes components not recommended for habitual consumption, such as sugar-sweetened beverages, added sugars and processed meats. The maximum score was assigned when there was no consumption and decreases proportionally as intake increases, until the maximum tolerated limit of consumption when the minimum score of 0 was assigned. Online Supplemental Figure 2 shows how the scoring was conducted.

The Eat-Lancet PH recommendations include twenty food groups, which we adapted to fourteen components of three dimensions. The healthy without waste dimension included whole grains, vegetables, fruits, legumes, nuts and seeds, and unsaturated oils. Since the SFFQ used does not distinguish whole grains, we included high-fibre cereals, considered to be those with a carbohydrate-to-fibre ratio of 10:1^(26)^. The unhealthy in excess dimension included starchy tubers or vegetables, milk, yoghurt and cheese, chicken and other poultry, eggs, fish, and seafood. Red meat, added saturated fats and added sugars were included in the unhealthy dimension. For this index, each component is presented as a range of points from 0 to 10 for a total range of 0–140 points. For components of dimensions that include zero in the recommended intake range, we replaced the minimum intake value of 0 with one equivalence unit from the Mexican system of equivalents for the healthy without waste dimension^(27)^. For the unhealthy in excess dimension, we replaced zeroes with a minimum of half of one equivalence unit, since the maximum intake recommended was equal to or lower than one equivalence unit. This adaptation was necessary because the EAT-Lancet recommendations consider consumption ranges with minimum values different from zero only for fruits, vegetables and unsaturated oils. Finding individuals who consume only these three food groups is unlikely in Mexico, and that could lead to lower scores. Intakes were adjusted to 2500 kilocalories (kcal) to frame the EAT-Lancet PH recommendations.

The GBD index derived from optimal intake estimated by GBD includes fifteen food groups across three dimensions. The healthy dimension includes fruits, vegetables, legumes, high-fibre cereals, nuts and seeds, milk, fibre, Ca, and *n*-3 and *n*-6 fatty acids. The unhealthy in excess dimension includes only red meat, and the unhealthy dimension includes processed meats, sugar-sweetened beverages, trans-fatty acids and Na. Each component has a maximum score of ten, except for fruits, vegetables, legumes, fibre, nuts and seeds, and *n*-3 or *n*-6 fatty acids, which have characteristics in common and may be scored at a maximum of five points. The total score may range from 0 to 120 points. Consumption per component was not adjusted to total energy intake per d, as this index does not establish it.

The MDG index considers nine food groups, and we divided animal-based foods into two components: high-fat and low-fat. Animal-based foods were separated by fat content because the guidelines indicate that at least half of the portions of this component should be low-fat. Therefore, the index derived from these recommendations includes ten food groups and two dimensions. The healthy dimension includes fruits, vegetables, cereals, legumes, low-fat animal-based foods, low-fat dairy products and plain water. The unhealthy dimension includes high-fat animal-based foods, free sugars and foods rich in fats. Each component may range from 0 to 10 points, except for low-fat and high-fat animal-based foods, scored from 0 to 5 points. The MDG recommends the number of servings to be consumed per food group per d according to total daily energy intake and age. Suggested serving sizes are in pieces, cups or tablespoons. In this analysis, we adjusted servings consumed by energy intake and age group (Table 4).

The percentage of adherence was reported to make possible comparisons between indexes and their components. It was calculated as the score obtained divided by the maximum possible score and multiplied by 100. For all three indexes, a higher percentage of adherence to recommendations indicates higher dietary quality.

### Sociodemographic indicators

Self-reported information on sex, age, student/non-student status, housing characteristics and household assets was obtained through a sociodemographic questionnaire. The student status variable was selected as a proxy of a set of social and family conditions related to school dropout and related to health and dietary quality^(28)^. These conditions include ethnicity, parental education, parental involvement in their children’s education and health, substance use, academic performance, etc.^(29)^ Also, it was included because a public policy in elementary and junior high schools was implemented in 2010. This strategy sought to restrict the availability of low-nutrient foods to the students. A household assets index was estimated using the housing characteristics (wall, floor and roof materials, toilet, drain, etc.) and ownership of durable assets (car, TV, radio, refrigerator, etc.) through a principal component analysis. The score of the first component was categorised into tertiles: tertile 1 as the lowest household asset level and tertile 3 as the highest. This variable was already available in ENSANUT databases.

Dwelling area: rural <2500 inhabitants and urban ≥2500 inhabitants.

Geographical region: North (Baja California, Baja California Sur, Coahuila, Chihuahua, Durango, Nuevo León, Sonora, Tamaulipas), Central (Aguascalientes, Colima, Guanajuato, Jalisco, Michoacán, Morelos, Nayarit, Querétaro, Mexico State, San Luis Potosí, Sinaloa, Zacatecas, and Mexico City), and South (Campeche, Chiapas, Guerrero, Hidalgo, Oaxaca, Puebla, Tlaxcala, Quintana Roo, Tabasco, Veracruz, Yucatán)^(17)^.

### Statistical analysis

Due to sample losses of over 10 % in three of the four ENSANUT surveys due to the data cleaning process, the weighted distribution of sex, age, dwelling area and region of the analysis samples was compared with the closest census or intercensal survey conducted by the Mexican National Institute of Statistics, Geography, and Informatics (INEGI)^(30–33)^. We found differences between the 2006 and 2012 surveys, so to mimic the observed distribution, the sampling weights were calibrated^(34)^ by sex, age, dwelling area, and region in 2006 and by sex and age in 2012.

Sociodemographic characteristics were described as percentages or means with 95 % CI, and the percentage of adherence to dietary indexes was described by 50, 25 and 75th percentiles as estimated by quantile regression models. Changes in dietary quality over time were evaluated using quantile regression models to estimate the medians of adherence measured by each index as a function of the survey year, total daily energy intake, age, sex, dwelling area, region, household assets level and student/non-student status; those covariates were chosen according to the dietary quality conceptual framework and the Mexican context about health public policies^(35)^. In addition, trends in dietary quality were estimated with stratified quantile regression models for each sociodemographic indicator adjusted by the other covariables; these coefficients are displayed graphically through forest plots. CI and *P* values were adjusted using Bonferroni correction, according to the number of regression models performed (see online supplementary material, Supplemental Table 2). All analyses were performed in Stata 16.0, and the complex sample design was considered.

## Results

After calibrating the sampling weights, sex, age, dwelling area and geographical region distributions were similar to the closest census or intercensal survey. Table 1 shows that the mean age of sampled adolescents was 15 years, the proportion of males was 51 % for all surveys, approximately three of every four were from urban localities, and nearly half lived in the central region of Mexico. The proportion of adolescents attending school was about 75 %, with 2016 being the highest (78·4 %). In 2012 and 2016, a lower proportion of adolescents were in the lowest household assets tertile (26·4 and 24 %, respectively) than in 2006 (31·9 %) and 2018 (33·5 %). The median of total daily energy intake was lower in 2012 (1634 kcal), while the highest was observed in 2016 (1978 kcal) (Table 1).

**Table 1:** Sample characteristics by survey

Characteristic	2006‡		2012§		2016||		2018¶		
	%	95 % CI	%	95 % CI	%	95 % CI	%	95 % CI	*P* value for differences
Age (years)*	15·4	15·3, 15·5	15·5	15·4, 15·6	15·3	15·2, 15·5	15·2	15·1, 15·4	0·0320
Sex									
Male	51·0	49·4, 52·7	51·1	47·7, 54·4	51·1	48·2, 54·0	51·2	49·0, 53·4	0·9998
Female	49·0	47·3, 50·6	48·9	45·6, 52·3	48·9	46·0, 51·8	48·8	46·6, 51·1	
Dwelling area									
Rural	25·2	22·8, 27·8	26·2	23·5, 29·1	27·3	23·6, 31·4	25·1	22·7, 27·5	0·7660
Urban	74·8	72·3, 77·2	73·8	70·9, 76·5	72·7	68·6, 76·5	75·0	72·5, 77·3	
Geographical region									
North	17·7	16·0, 19·6	21·5	19·4, 23·7	17·7	13·8, 22·5	19·3	17·7, 21·0	0·2008
Central	49·4	46·3, 52·5	47·6	44·4, 50·7	48·0	42·8, 53·1	46·9	44·2, 49·6	
South	32·9	30·1, 35·9	30·9	28·2, 33·8	34·3	29·7, 39·4	33·8	31·5, 36·2	
School attendance									
Non-student	26·0	24·3, 27·7	24·9	22·3, 27·7	21·6	19·0, 24·4	22·7	20·7, 24·9	0·0233
Student	74·1	72·3, 75·7	75·1	72·4, 77·7	78·4	75·6, 81·1	77·3	75·1, 79·3	
Household assets tertile									
Low	31·9	29·7, 34·1	26·4	23·7, 29·2	24·0	20·7, 27·7	33·5	31·3, 35·8	<0·001
Medium	33·3	31·3, 35·4	32·8	29·8, 35·9	33·2	29·7, 36·8	35·0	32·8, 37·2	
High	34·8	32·5, 37·2	40·8	37·6, 44·2	42·8	38·7, 47·1	31·5	29·3, 33·7	
Total energy intake (kcal/d)†	1633·8	1262·0, 2105·8	1908·9	1442·4, 2392·2	1977·7	1497·8, 2584·2	1756·3	1348·0, 2323·6	<0·001

* Mean.

† 50 and 25, 75th percentiles.

‡ Sample = 7266, expanded population = 18 489 041 Mexican adolescents (12–19 years).

§ Sample = 1902, expanded population = 18 125 466 Mexican adolescents (12–19 years).

|| Sample = 2335, expanded population = 15 472 479 Mexican adolescents (12–19 years).

¶ Sample = 5017, expanded population = 17 490 087 Mexican adolescents (12–19 years).

Table 2 shows the unadjusted median of the percentage of adherence for the PH index as 40 %. The components with the highest adherence to recommendations were fruits (81–85·6 %), high-fibre cereals and vegetables (51·2–69·1 %), dairy products and chicken/poultry (62·8–99·9 %), and saturated fats (55·9–70·3 %). In contrast, the components with the lowest adherence (0 %) were nuts and seeds, eggs, red meat, and added sugars. For the GBD index, the median adherence was between 33·9 and 37 %. Components with the highest adherence were high-fibre cereals (100 %), fibre (81·2–92·3 %), and Ca (65·9–77·1 %), while nuts and seeds, milk, red meat, processed meats, and sugar-sweetened beverages showed adherence of 0 % (Table 3). For the MDG index, the median adherence was 36·1 to 39·2 %. The only component that showed high adherence was cereals (100 %), whereas components with the lowest adherence were legumes (4–5·7 %), low-fat dairy products (20·3–28·3 %), vegetables (26·4–34·4 %), and high-fat animal-based foods and added sugars with 0 to 3·7 % adherence (Table 4).

**Table 2:** Adherence of Mexican adolescents to the Planetary Health index, by survey year

Index components	Criteria for		Adherence percentage							
	Perfect adherence*	Non-adherence	2006		2012		2016		2018	
			Median	25, 75th percentiles	Median	25, 75th percentiles	Median	25, 75th percentiles	Median	25, 75th percentiles
Total			39·8	33·1, 46·6	39·8	33·1, 46·6	41·5	34·9, 48·2	40·4	33·6, 47·3
**Healthy without waste components**										
High-fibre cereals	232 g/d	0 or ≥85th percentile between upper limit of recommendation and maximum intake in sample	54·9	23·6, 78·8	54·9	23·6, 78·8	60·1	31·8, 82·6	57·1	29·6, 81·3
Vegetables	200–600 g/d		51·2	31·7, 82·5	51·2	31·7, 82·5	69·1	38·5, 100	53·9	33·3, 88·8
Fruits	100–300 g/d		81·0	31·2, 100	81·0	31·2, 100	90·4	47·2, 100	85·6	40·1, 100
Legumes†	35–100 g/d		23·7	10·4, 46·8	23·7	10·4, 46·8	28·4	11·0, 62·7	28·0	12·6, 57·3
Nuts and seeds	10–75 g/d		0·0	0·0, 0·8	0·0	0·0, 0·8	0·0	0·0, 0·2	0·0	0·0, 1·1
Unsaturated oils	20–80 g/d		37·2	19·5, 69·3	37·2	19·5, 69·3	39·0	21·4, 70·7	43·9	22·9, 75·5
**Unhealthy in excess components**										
Tubers	25–100 g/d	0 or > upper limit of the recommendation	23·8	2·6, 63·5	23·8	2·6, 63·5	21·5	4·1, 64·2	20·9	6·0, 68·4
Dairy products	125–500 g/d		62·8	0·1, 100	62·8	0·1, 100	85·9	2·7, 100	99·9	21·2, 100
Chicken/poultry	15–58 g/d		99·7	1·9, 100	99·7	1·9, 100	67·3	0·0, 100	66·0	0·0, 100
Eggs	7–25 g/d		0·0	0·0, 27·3	0·0	0·0, 27·3	0·0	0·0, 83·9	0·0	0·0, 37·6
Fish and shellfish	14–100 g/d		5·1	0·9, 75·1	5·1	0·9, 75·1	4·5	0·8, 54·9	5·9	2·1, 63·2
**Unhealthy components**										
Red meat	0	28 g/d	0·0	0·0, 53·8	0·0	0·0, 53·8	0·6	0, 65·6	0·0	0·0, 41·4
Saturated fats	0	11·8 g/d	70·3	31·5, 90·2	70·3	31·5, 90·2	60·3	23·1, 82·3	55·9	15, 81·8
Added sugars	0	31 g/d	0·0	0·0, 0·0	0·0	0·0, 0·0	0·0	0·0, 0·0	0·0	0·0, 0·0

* Intermediate adherences were estimated according to equations presented in online supplementary material, Supplemental Fig. 2.

† Dried and raw weight.

**Table 3:** Adherence of Mexican adolescents to the Global Burden of Disease index, by survey year

Index components	Criteria for		Adherence percentage							
	Perfect adherence*	Non-adherence	2006		2012		2016		2018	
			Median	25, 75th percentiles	Median	25, 75th percentiles	Median	25, 75th percentiles	Median	25, 75th percentiles
Total			35·2	29·6, 41·7	34·6	29·2, 40·7	37·0	31·8, 42·2	33·9	28·8, 39·5
**Healthy Components**										
Fruits†	≥250 g/d	0	38·0	11·1, 78·1	50·4	20·1, 98·2	47·6	20·8, 95·0	34·7	13·1, 70·8
Vegetables†	≥360 g/d	0	18·5	10·8, 31·5	30·6	17·1, 48·7	31·9	16·2, 55·8	21·3	12·4, 37·3
Legumes†	≥60 g/d	0	9·1	3·7, 18·7	16·2	5·5, 36·9	13·9	5, 31·8	12·3	5·1, 25·2
High-fibre cereals	≥125 g/d	0	100	67, 100	100	57·5, 100	100	88·7, 100	100	72·4, 100
Nuts and seeds†	≥21 g/d	0	0·0	0·0, 0·4	0·0	0·0, 0·3	0·0	0·0, 0·0	0·0	0·0, 0·4
Milk	≥435 g/d	0	0·1	0·0, 24·5	0·3	0·0, 28·3	0·3	0·0, 26·7	0·5	0·0, 28·7
Fibre†	≥24 g/d	0	81·2	59·9, 98·8	89·8	64·3, 100	92·3	68·8, 100	86·6	61·4, 100
Ca	≥1·25 g/d	0	65·9	48·6, 91·6	74·2	52·5, 95·5	77·1	54·3, 95·7	67·4	49·2, 90·0
*n*-3 fatty acids†	≥250 mg/d	0	19·7	10·7, 40·6	20·3	11·5, 35·7	22	11·9, 49·3	20·9	11·6, 42·9
*n*-6 fatty acids†	≥11 % of energy intake	0	31·9	24·7, 39·7	32·9	26·5, 39·7	31·3	23·8, 38·8	32·9	26·3, 40·4
**Unhealthy in excess components**										
Red meat	18–27 g/d	0 or >27 g/d	0·0	0·0, 64·3	0·0	0·0, 49·9	3·3	0·0, 63·8	0·0	0·0, 57·1
**Unhealthy components**										
Processed meats	0	>2 g/d	0·0	0·0, 98·1	0·0	0·0, 0·0	0·0	0·0, 10·1	0·0	0·0, 0·0
Sugar-sweetened beverages	0	>3 g/d	0·0	0·0, 0·0	0·0	0·0, 0·0	0·0	0·0, 0·0	0·0	0·0, 0·0
Trans-fatty acids	0	>0·5 % of energy intake	18·4	0·0, 57·8	3·5	0·0, 49·8	21·6	0·0, 59·5	20·3	0·0, 54·1
Na	0	>3 g/d	37·5	12·1, 56·6	23·3	1·3, 45·9	28·5	6·0, 47·2	33·6	9·0, 51·3

* Intermediate adherences were estimated according to equations presented in online supplementary material, Supplemental Fig. 2.

† Maximum adherence is based on a maximum score of 5 points.

**Table 4:** Adherence of Mexican adolescents to the Mexican Dietary Guidelines index, by survey year

	Number of portions for								Adherence percentage							
Adherence (%)	Perfect adherence, by age*,†				Non-adherence, by age*,†				2006		2012		2016		2018	
	12 y	13–15 y	16–18 y	19 y	12 y	13–15 y	16–18 y	19 y	Median	25, 75th percentiles	Median	25, 75th percentiles	Median	25, 75th percentiles	Median	25, 75th percentiles
Total									37·4	31·2, 43·9	37·4	31·1, 43·7	39·2	33·3, 46·3	36·1	30·2, 43·0
**Healthy components**																
Vegetables	≥3	≥3	≥4	≥3	0				26·4	15·4, 45·4	34·4	19·8, 58·8	31·0	17·4, 56·8	26·7	15·8, 45·6
Fruits	≥2	≥3	≥3	≥3	0				38·2	11·6, 78·4	50·3	21·2, 93·9	48·9	23·1, 88·6	40·6	17·1, 78·1
Cereals	≥7·5	≥8	≥11	≥8	0				100	95·1, 100	100	81·5, 100	100	90·8, 100	100	79·4, 100
Legumes	≥2	≥2	≥2·5	≥2	0				4·0	1·7, 7·8	5·7	2·1, 12·4	4·7	1·9, 10·7	4·7	2·1, 9·6
Low-fat animal-based foods	≥3	≥3·5	≥3·5	≥3·5	0				39·3	20·8, 63·8	36·5	16·9, 58·6	38·9	19·5, 66·7	46·5	25·5, 76·5
Low-fat dairy products	≥2				0				26·3	8·4, 64·6	28·3	5·8, 67·7	20·3	3·5, 60	23·5	6·1, 57·2
Plain water	≥8				0				39·5	19·7, 69·3	37·9	14·4, 73·6	52·2	29·5, 90·2	52·3	25·4, 88·3
**Unhealthy components**																
High-fat animal-based foods	0				≥1·5	≥1·75	≥1·75	≥1·75	0·0	0·0, 39	0·0	0, 29·2	3·7	0, 41·1	0·0	0·0, 28·1
Added Sugars	0				≥2	≥2	≥3	≥2	0·0	0·0, 0·0	0·0	0·0, 0·0	0·0	0·0, 0·0	0·0	0·0, 0·0
Foods rich in fats	0				≥4	≥5	≥5	≥5	41·0	1·9, 66·8	26·4	0·0, 53·8	34·3	0·0, 60·2	17·7	0·0, 48

* Intermediate adherences were estimated according to equations presented in online supplementary material, Supplemental Fig. 2.

† Total energy intake by age group: 12 years = 1800 kcal/d, 13–15 years = 2000 kcal/d, 16–18 years = 2400 kcal/d, 19 years = 2000 kcal/d.

**Fig. 1:**
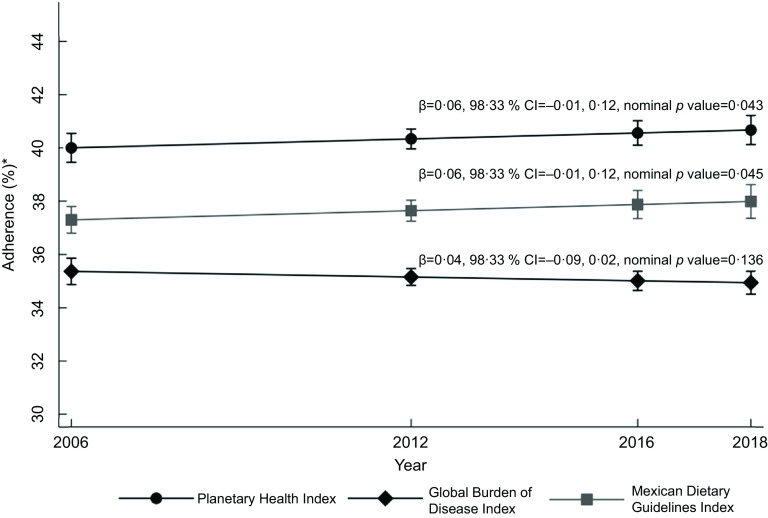
Trends in dietary quality in Mexican adolescents *Median estimated through quantile regression models as function of survey year, age, sex, dwelling area, geographical region, household assets index, student/non-student status and total energy intake per d. Confidence level = 98·33 % and *P* value for significance <0·017 with Bonferroni correction

Figure 1 shows the adjusted medians of the percentage of adherence to these three indexes. The dietary quality of Mexican adolescents was estimated at below 41 %, with the GBD index demonstrating the lowest (34·9–35·4 %) and the PH index the highest (40–40·6 %). Over time, no important change in adherence was found for any of the three indexes (Fig. 1).

**Fig. 2:**
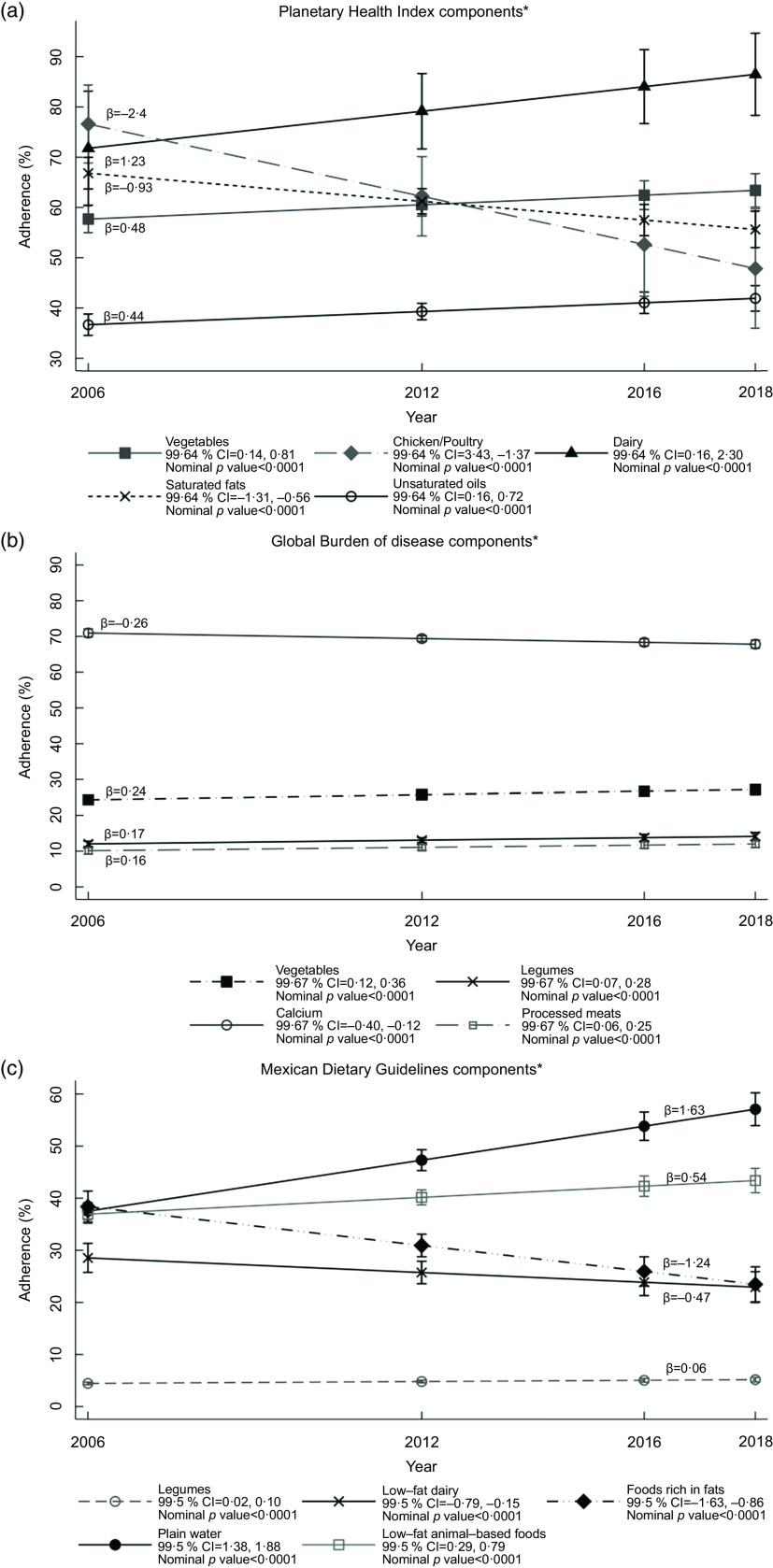
Trends in adherence of Mexican adolescents to recommendations, by component of dietary quality index *Only components with relevant trends are shown. Median adherences estimated through quantile regression models as function of survey year, age, sex, dwelling area, geographical region, household assets tertile, student/non-student status and total energy intake per d. *P* values and CI were adjusted using Bonferroni correction. Trends in Fig. 2(a) at 99·64 % confidence level, *P* value for significance <0·0036. Trends in Fig. 2(b) at 99·67 % confidence level, *P* value for significance <0·0033. Trends in Fig. 2(c) at 99·50 % confidence level, *P* value for significance <0·005

Although shifting trends in total adherence for each index over time were not found, some components showed substantial changes from 2006 to 2018 (Fig. 2(a–c)). For the PH index, components that showed significant increases in the percentage of adherence to recommendations were dairy products (*β* = 1·23, +20·5 %), unsaturated oils (*β* = 0·44, +14·3 %) and vegetables (*β* = 0·48, +9·8 %). On the other hand, reductions over time were demonstrated for chicken/poultry (*β* = –2·4, –37·6 %) and saturated fat (*β* = –0·93, –16·8 %) (Fig. 2(a)). In the GBD index, a negative trend over time was found for Ca (*β* = –0·26, –4·4 %), whereas slight increases in adherence were found for vegetables (*β* = 0·24, +11·6 %), legumes (*β* = 0·17, +17·4 %) and processed meats (*β* = 0·16, +18·6 %). It is important to emphasise that all groups mentioned revealed adherences below 25 % (Fig. 2(b)). Finally, Fig. 2(c) shows that the MDG index components showing increases in adherence from 2006 to 2018 were plain water (*β* = 1·63, +52 %), low-fat animal-based foods (*β* = 0·54, +17·5 %) and legumes (*β* = 0·06, +16·2 %), while foods rich in fats (*β* = –1·24, –38·8 %) and low-fat dairy products (*β* = 0·47, –19·7 %) showed reductions.

**Fig. 3:**
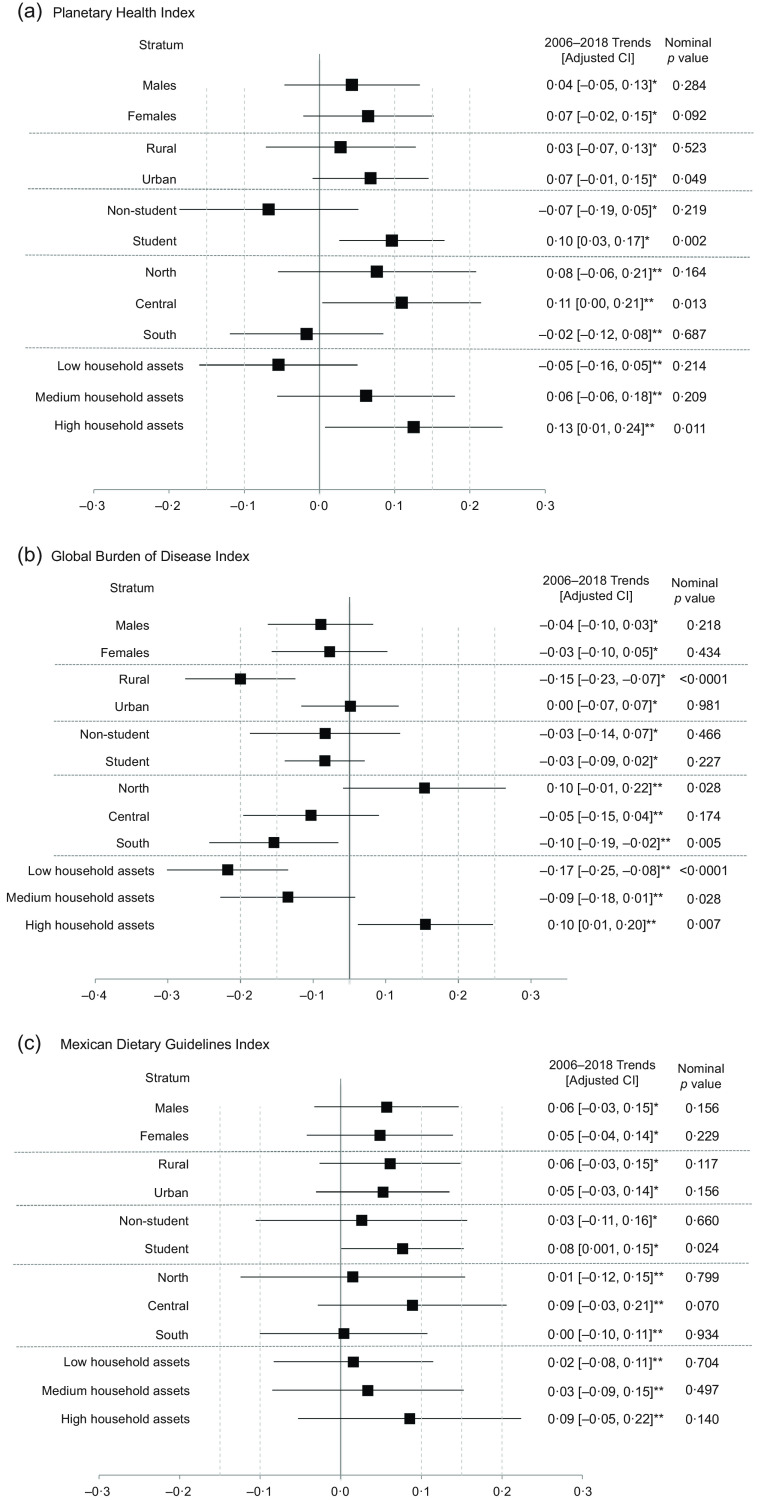
Trends in dietary quality in Mexican adolescents, by sociodemographic indicator. *Adjusted confidence level = 97·5 %, adjusted *P* value for significance <0·025 with Bonferroni correction. **Adjusted confidence level = 98·33 %, adjusted *P* value for significance <0·017 with Bonferroni correction

Figure 3(a) displays a trend analysis of total adherence to each index by sociodemographic indicator, which shows an increase from 2006 to 2018 in PH adherence in adolescents who are students and those who live in the central region of Mexico and adolescents who have a higher household assets condition. For the GBD index, positive trends were found in adolescents with higher household assets condition. In contrast, adolescents from rural areas, from the South and with low household assets showed negative trends in adherence over time (Fig. 3(b)). Regarding the MDG index, adolescents who attended school showed increased adherence from 2006 to 2018 (Fig. 3(c)).

## Discussion

In this analysis, we found that Mexican adolescents have low dietary quality, whether based on the recommendations for PH by EAT-Lancet, the optimal intake estimated by GBD studies or the 2015 MDG (<40 % adherence). This scenario is worrisome given the health effects of a low-quality diet, such as obesity, nutritional deficiencies and diet-associated chronic diseases, among others^(1)^.

### Differences and similarities between indices to estimate dietary quality

Despite differences in dimensions, components included and intake recommendations between indexes, common elements may elucidate the low adherences found. Low adherence (due to high intake) was identified for unhealthy components, such as added sugars in the MDG and PH or sugar-sweetened beverages in the GBD index and also in red meat, processed meats in the PH and GBD, high-fat animal-based foods, or foods rich in fats in MDG index. Conversely, low adherence (due to low intake) was revealed in healthy or healthy without excess components such as legumes, vegetables, nuts and seeds, and unsaturated oils. Moreover, the higher adherence in the fruits component in the PH index than those found for MDG and GBD indexes may be attributed to the PH index’s lower intake recommendation (100–300 *v*. ≥250 g/d or ≥2–3 portions). Consequently, a higher proportion of adolescents may have fruit consumption within the recommended range (see online supplementary material, Supplemental Tables 3–5).

### Comparison of dietary quality findings with other studies

Directly comparing our findings with other reports is challenging due to limited available information and variations in foods included in each component, consumption criteria and scoring methods employed by different indexes. Nonetheless, there are reports of low adherence to legumes, vegetables and Na consumption among individuals under the age of 20 years in the USA^(36)^ (approximately 50 % for vegetables, between 40 and 50 % for Na, and below 20 % for legumes), as well as low legume adherence in those aged 2–19 years in Mexico (<20 %)^(11)^. Our sample shows lower adherence to added sugars and sugar-sweetened beverages compared with other populations, aligning with the documented high intake of sugar-sweetened beverages in the Mexican population^(37,38)^. Conversely, we observed higher adherence in high-fibre cereals (mainly tortilla)^(36,39)^. Castellanos *et al.*, in their adaptation of the EAT-Lancet index in Mexican adults with 24-h recall, found similar results regarding food groups where consumption falls far from the recommendation^(40)^.

### Overall trends in dietary quality

Regarding trends over time, we did not identify important changes in total adherence using any of the three assessed approaches. Although some components of the GBD index showed relevant trends, these were small, which may explain why total adherence to the GBD index was not significant. It is important to highlight that adherence differed among the three indexes. The PH showed the highest adherence and the GBD the lowest; the latter contains more components with adherence of zero or below 50 %. One possible explanation for these results is that the GBD recommendations are not framed by daily total energy intake, possibly assigning low scores to individuals with lower intakes of components in the healthy dimensions, even if those could be appropriate in proportion to their overall diet intake. The same principle applies to individuals with high intakes of components of the unhealthy dimension. Therefore, by including total daily energy intake in the framing of the optimal intake, GBD estimations could enhance their applicability as intake recommendations.

### Trends in dietary quality by components

Liu *et al.* ^(36)^ reported increased adherence to HEI-2015 and American Heart Association (AHA) recommendations for vegetables, cereals, legumes, and dairy products between 1999 and 2016 in the 2–19 years age group in the USA; they also found decreased adherence in saturated fat, which could be compatible with our results. Notably, a pronounced reduction observed in chicken/poultry adherence through the PH index could be linked to an increase in consumption of this food group over time, exceeding the upper limit of intake recommendations in the healthy without waste component dimension. This is evident in the proportion of adolescents who exceeded recommendations in 2018 (23·6 %, 95 % CI: 21·6–25·7 %) in comparison with the 2016 survey (16·3 %, 95 CI %:13·6–19·5 %) (see online supplementary material, Supplemental Table 2).

An essential component to highlight is legumes, as both the GBD and MDG indexes showed an increase in adherence to recommendations. However, the percentage of adherence remains alarmingly low, with a high proportion of adolescents reporting intakes below the recommendations outlined by any of the three indexes (see online supplementary material, Supplemental Tables 2–4). Legumes form part of the traditional Mexican diet, making the reported low intake among adolescents a cause for concern. This concern stems from the fact that higher legume intake is related to lower CVD^(41)^ and legumes are a sustainable source of proteins^(9)^.

### Trends in dietary quality by sociodemographic stratum

Analysing trends in dietary quality across sociodemographic strata reveals a noteworthy pattern. There is an observed increase in adherence for adolescents with high household assets level or those attending school. Conversely, adolescents from rural areas and with low household assets levels showed negative trends in adherence over time. These findings are consistent with previous reports associating higher dietary quality index scores with higher costs^(42,43)^. Additional trends highlighted by Batis *et al.* indicate a lower probability of fruit and vegetable consumption in populations from rural areas compared with urban areas^(44)^. Furthermore, higher scores in refined grains, Na and saturated fats were reported in the rural population^(25)^.

### Trends in dietary quality by geographical region

Our trend results by geographical region align with 2018 reports showing that adolescents from the South, which have more inhabitants in poverty than other Mexican regions, were more likely to have higher consumption of sugar-sweetened beverages and lower consumption of legumes and vegetables than those in the North^(21,44)^. This evidence supports the premise that populations from North and Central Mexico or urban areas have better economic and social environments, enabling access to diets of higher quality and diversity as compared with their counterparts in South or rural areas^(44)^. However, further research is needed to identify which components of these indexes contribute to the observed differences in dietary quality trends between urban/rural areas and among different regions, as our analysis only provides a national-level breakdown.

### Trends in dietary quality by school attendance

Social and familial factors can impact school attendance and, in turn, the dietary quality of adolescents^(28,29)^. Under this assumption, it is likely that our findings reflect that adolescents who had dropped out of school could have a context characterised by low parental education level, lack of family support, substance use and poor academic performance, among others^(29)^. These factors are also associated with lower dietary quality^(28)^. Our results were consistent with the expected direction for PH and MDG indexes, where adolescents who attended school showed an increase in dietary quality over time.

That finding could also be related to the guidelines for the sale of foods and beverages in Mexican primary and secondary schools. These guidelines seek to reduce the availability of food with low nutritional quality within school facilities^(35)^. Although the evidence shows that overall schools have low compliance with the guidelines^(45)^, it is likely that the limited compliance helps students to improve their diets in comparison with adolescents who do not attend schools^(46)^. Future studies will be needed to corroborate this hypothesis.

### Trends by sociodemographic characteristics: the particular case of the Mexican Dietary Guidelines index

For the MDG index, trends by sociodemographic characteristics exhibited wider CI, making it difficult to detect relevant trends. This may be because the recommendations for this population do not allow sufficient differentiation of the study sample. In other words, intake recommendations for the healthy components seem to be remarkably high in comparison with the number of portions consumed regularly by Mexican adolescents. Conversely, for the unhealthy components, the number of portions recommended seems to be very low in comparison with regular consumption in this population. As shown in online supplementary material, Supplemental Table 4, most adolescents are classified either below the recommendations in healthy components, except for cereals, or exceed the tolerable intake in the unhealthy components. Recently, following a review of these guidelines by a group of experts, the Mexican Ministry of Health published the new ‘healthy and sustainable dietary guidelines’^(47)^. Those seek to translate the intake recommendations of the EAT-Lancet Commission for the Mexican population. Future research will be needed to assess the impact of these new Mexican guidelines on health indicators.

### Limitations and strengths

This analysis has some limitations, mainly derived from the method used to collect dietary information^(48)^; the SFFQ is a closed list of food items, limiting adequate consumption estimations of some food groups such as whole grain, nuts and seeds, ultra-processed foods, and unusual foods. This limitation may lead to an underestimation of fibre, fatty acids and Na intakes. Another limitation is the information available in food composition tables, which classify maize tortilla as a high-fibre cereal, although this may not be true of industrialised tortillas^(49)^. This could overestimate adherence for cereals, as was observed in a sensitivity analysis (data not shown).

On the other hand, our analysis has key strengths. It documents dietary quality derived from nationally representative samples of the adolescent population using comparable methodologies from 2006 to 2018. In addition, it is among the first to assess dietary quality using three different approaches and report changes over time for the adolescent age group. Our results lay the groundwork for multiple future studies on this topic.

### Conclusions

We can conclude through three approaches that dietary quality in the Mexican adolescent population was very low from 2006 to 2018. Moreover, according to the PH, it was not sustainable due to the high consumption of food groups with production systems negatively impacting the environment^(9)^. Adolescents showed low adherence to recommendations for components harmful to health (sugars, saturated fats), indicating significant risks of developing diet-associated chronic diseases in the early stages of their lives^(3)^. Considering the current epidemiological and environmental context, further research and strategies will be crucial to promote diets that lead to an adequate state of human and environmental health.

It is critical to study the relationship of these indexes with health outcomes and/or biomarkers in this age group to adjust the focus of recommendations. It is advisable to design creative actions to increase nutritional literacy in adolescents, increase the regulation and monitoring of foods and beverages that can be sold in school or work facilities, maintain and strengthen tax measures on energy-dense foods and sugar-sweetened beverages, and promote strategies for local trade of fruits, vegetables, legumes, and other healthy food groups.

## Supporting information

Gaona-Pineda et al. supplementary material Gaona-Pineda et al. supplementary material

## Supplementary material

For supplementary material accompanying this paper, visit https://doi.org/10.1017/S1368980024000648.
