# Supplementary material for: Three approaches to assessing dietary quality in Mexican adolescents from 2006 to 2018 with data from national health and nutrition surveys
Source: Public Health Nutr. 2024 Mar 11;27(1):e97. doi: 10.1017/S1368980024000648 (PMC10993068; doi:10.1017/S1368980024000648)
Supplement: Gaona-Pineda et al. supplementary material [file S1368980024000648sup001.docx]

**Supplementary Material**

**Supplementary Table 1. Foods and beverages included in each food group component, by dietary quality index**

| **Food Group Components** | **Planetary Health Index** | **Global Burden of Disease Index** | **Mexican Dietary Guidelines** |
| --- | --- | --- | --- |
| High-fiber cereals (fiber/carbohydrates >0.1) | Tortilla, corn, whole grain bread, popcorn | Tortilla, corn, whole grain bread, popcorn, whole grain breakfast cereals | - |
| Cereals | - | - | Rice, sweet potato, breakfast cereal, corn, cookies, all types of bread, wheat pasta, wheat or corn flour, instant soup, all types of tortilla |
| Vegetables | Broccoli, cauliflower, zucchini, *chayote*, poblano peppers, cabbage, green beans, leafy greens, tomato, lettuce, *nopal*, cucumber, carrot, peas, onion, mushrooms, leak, *jicama*, canned or frozen vegetables | | |
| Fruits | Peach, strawberry, guava, mango, apple, pear, melon, watermelon, orange, papaya, pineapple, banana, grapefruit, grapes, *capulín*, plum, other fruits as ingredients of preparations included in the food frequency questionnaire (FFQ) | Peach, strawberry, guava, mango, apple, pear, melon, watermelon, orange, papaya, pineapple, banana, grapefruit, grapes | Peach, strawberry, guava, mango, apple, pear, melon, watermelon, orange, papaya, pineapple, banana, grapefruit, grapes, *capulín*, plum, other fruits as ingredients of preparations included in the FFQ |
| Legumes | Beans, lentils, broad beans, chickpeas | | |
| Nuts and seeds | Peanuts, fava beans, *pepitas*, almonds from ingredients of preparations included in the FFQ | | - |
| Unsaturated oils | Corn, sunflower, and canola oils as ingredients of preparations included in the FFQ | - | - |
| Tubers | Potato, sweet potato | - | - |
| Dairy | Whole, skim, or semi-skim milk, fresh/white cheese, cured/mature cheeses, whole or low-fat plain or flavored yogurt | - | - |
| Milk | - | Whole, skim, or semi-skim milk | - |
| Low-fat dairy | - | - | Skim or semi-skim milk, fresh/white cheese and low-fat yogurt |
| Chicken/poultry | Any piece of chicken or other poultry | - | - |
| Eggs | Boiled, fried, or scrambled egg | - | - |
| Fish and shellfish | Fresh or dried fish, tuna, sardines, any other seafood | - | - |
| Red meats | Beef, pork | Beef, pork | - |
| Processed meats | - | Ham, sausage | - |
| Low-fat animal-based foods | - | - | Seafood (shrimp, oysters, etc.), tuna and sardines (in tomato, water, or oil), lean poultry, pork, beef or dried beef, fresh or dried fish |
| High-fat animal-based foods | - | - | Egg, semi-fatty or fatty chicken, pork, beef, sausage |
| Saturated fats | Butter, lard, bacon, milk cream | - |  |
| Foods rich in fats | - | - | Vegetable oils, avocado, butter, lard, bacon, milk cream, mayonnaise, vegetable shortening |
| Added sugars | Sweeteners added to milk, tea, coffee, or desserts, condensed milk, sugars from sodas or other industrialized beverages, natural or industrialized juices, candies, etc. | - | Sweeteners added to milk, tea, coffee, or desserts, condensed milk, sugars from sodas or other industrialized beverages, natural or industrialized juices, candies, etc. |
| Water | - | - | Plain water |
| Sugar-sweetened beverages | - | Industrialized juices, sodas, carbonated beverages, fruit drinks, etc. | - |


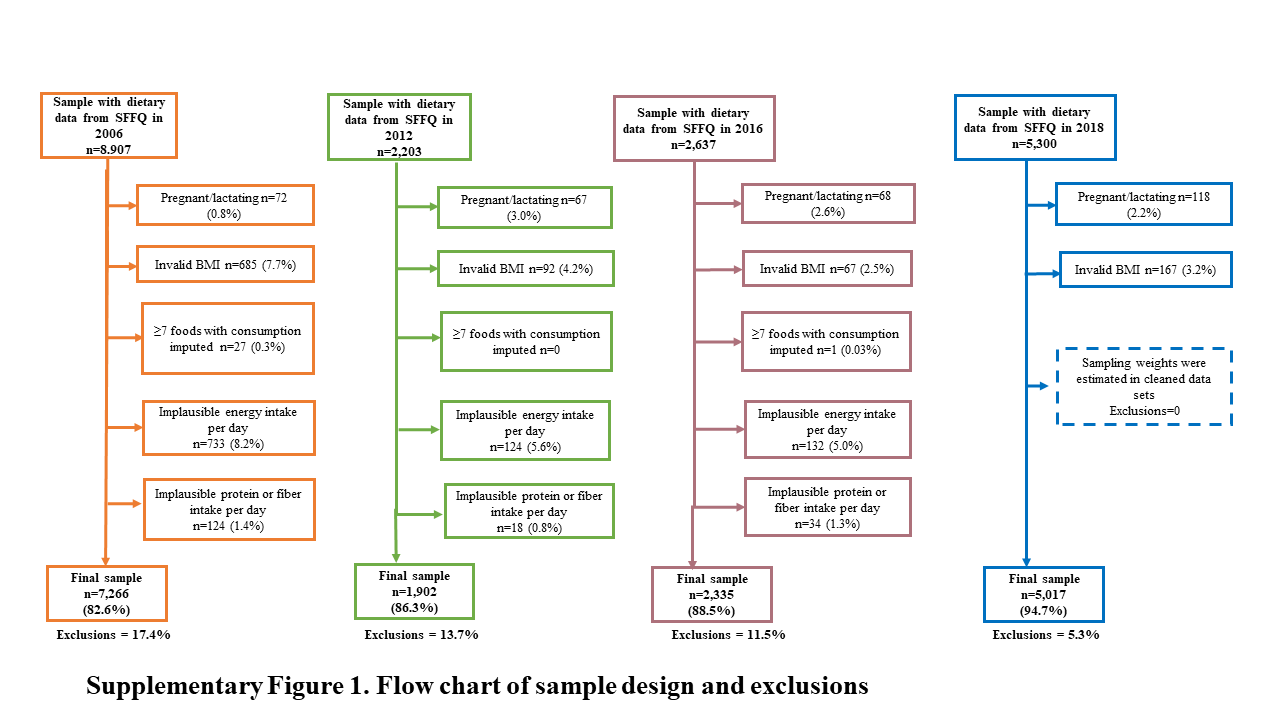


Supplementary Figure 2. Scoring by component dimension

| 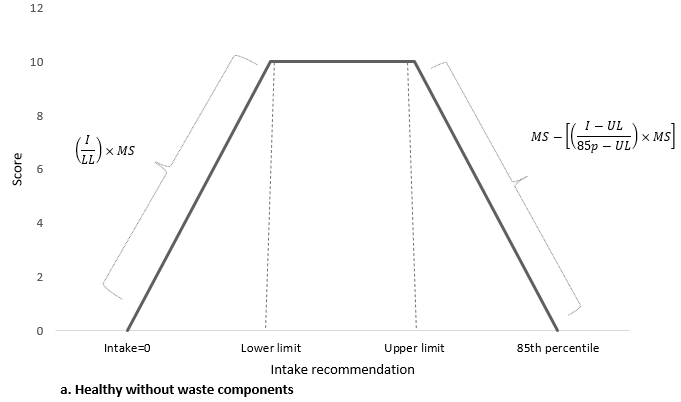 | 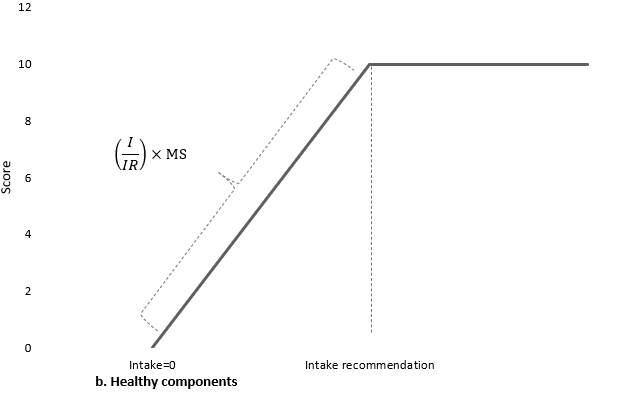 |
| --- | --- |
| 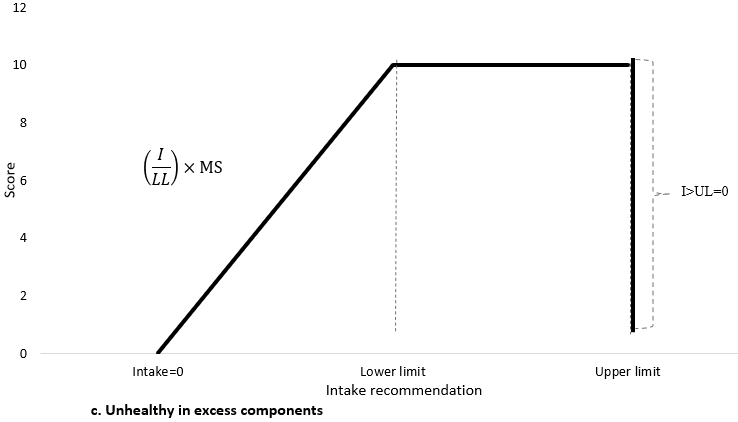 | 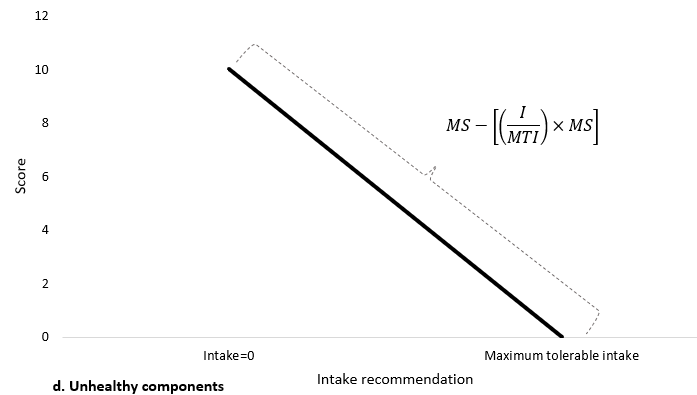 |
| Where: I=Intake, LL=Lower limit of intake recommendation, UL= Upper limit of intake recommendation, 85p=85^th^ percentile between UL and maximum intake registered in sample, MS=Maximum score, IR=Intake recommendation, MTI=Maximum tolerable intake. | |

**Supplementary Table 2. Bonferroni correction used in statistical analysis, *p* values for significance and confidence levels.**

| Analysis | *p* value for significance | Confidence level |
| --- | --- | --- |
| Trends in dietary quality (Figure 1) | 0.05/3 = 0.017 | 98.33% |
| Trends in dietary quality by Planetary Health Index components (Figure 2a) | 0.05/14 = 0.0036 | 99.64% |
| Trends in dietary quality by Global Burden of Disease Index components (Figure 2b) | 0.05/15 = 0.0033 | 99.67% |
| Trends in dietary quality by Mexican Dietary Guidelines Index components (Figure 2c) | 0.05/10 = 0.005 | 99.50% |
| Trends by sex, dwelling area and student status (Figure 3a, 3b, 3c) | 0.05/2=0.025 | 97.50% |
| Trends by region and household assets strata (Figure 3a, 3b,3c) | 0.05/3 = 0.017 | 98.33% |

**Supplementary Table 3. Distribution of the Mexican adolescent population according to component intake recommendation of the Planetary Health Index, by survey year**

| **Intake vs. Recommendation Category** | 2006 | | 2012 | | 2016 | | 2018 | |
| --- | --- | --- | --- | --- | --- | --- | --- | --- |
|  | % | CI95% | % | CI95% | % | CI95% | % | CI95% |
| **High-Fiber Cereals** |  |  |  |  |  |  |  |  |
| No intake | 1.2 | 0.9, 1.6 | 2.4 | 1.7, 3.5 | 0.8 | 0.4, 1.5 | 0.6 | 0.4, 1 |
| Intake<recommendation | 46.9 | 44.6, 49.2 | 56.4 | 53.2, 59.6 | 46.4 | 42.6, 50.2 | 49.5 | 47.2, 51.8 |
| Intake=recommendation | 0.2 | 0.1, 0.4 | 0.3 | 0.1, 0.8 | 0.4 | 0.2, 1.1 | 0.3 | 0.1, 0.6 |
| Intake>recommendation | 44.3 | 42.2, 46.3 | 34.9 | 32, 37.9 | 45.7 | 42, 49.5 | 42 | 39.8, 44.3 |
| Intake>85^th^ p* | 7.5 | 6.5, 8.6 | 6 | 4.7, 7.6 | 6.7 | 5.2, 8.7 | 7.6 | 6.5, 8.8 |
| **Vegetables** |  |  |  |  |  |  |  |  |
| No intake | 0.1 | 0, 0.2 | 0 | 0, 0 | 0 | 0, 0 | 0 | 0, 0.2 |
| Intake<recommendation | 82.5 | 81, 83.8 | 66.9 | 63.9, 69.8 | 65.8 | 62.7, 68.9 | 78.2 | 76.3, 80.1 |
| Intake=recommendation | 16.5 | 15.2, 17.9 | 31.9 | 29.1, 34.9 | 32 | 28.9, 35.2 | 20.1 | 18.3, 22 |
| Intake>recommendation | 0.8 | 0.5, 1.3 | 0.9 | 0.5, 1.6 | 2.1 | 1.3, 3.2 | 1.4 | 0.9, 2.1 |
| Intake>85^th^ p* | 0.1 | 0, 0.4 | 0.3 | 0.1, 1.1 | 0.2 | 0.1, 0.4 | 0.3 | 0.1, 0.7 |
| **Fruits** |  |  |  |  |  |  |  |  |
| No intake | 6.4 | 5.5, 7.4 | 5.7 | 4.4, 7.5 | 3.5 | 2.3, 5.1 | 4.1 | 3.3, 4.9 |
| Intake<recommendation | 31.2 | 29.4, 33 | 27.2 | 24.6, 29.9 | 29.2 | 26.6, 32 | 36.4 | 34.3, 38.6 |
| Intake=recommendation | 37 | 35.1, 38.9 | 38.1 | 35.1, 41.3 | 41.6 | 38.4, 44.9 | 39.9 | 37.7, 42.2 |
| Intake>recommendation | 22 | 20.4, 23.6 | 24.3 | 21.6, 27.3 | 22.3 | 19.6, 25.3 | 17.1 | 15.5, 18.9 |
| Intake>85^th^ p* | 3.5 | 2.9, 4.1 | 4.7 | 3.4, 6.3 | 3.4 | 2.3, 5 | 2.4 | 1.9, 3.1 |
| **Legumes** |  |  |  |  |  |  |  |  |
| No intake | 3.7 | 3, 4.6 | 4.3 | 3.2, 5.7 | 3.4 | 2.4, 5 | 2.8 | 2.1, 3.6 |
| Intake<recommendation | 89.2 | 88, 90.3 | 81.3 | 79, 83.4 | 83.4 | 80.6, 85.8 | 88.7 | 87.2, 90 |
| Intake=recommendation | 6.9 | 6.1, 7.9 | 13.7 | 11.8, 15.8 | 12.4 | 10.3, 14.9 | 8 | 6.9, 9.2 |
| Intake>recommendation | 0.1 | 0.1, 0.2 | 0.6 | 0.4, 1.1 | 0.8 | 0.4, 1.7 | 0.4 | 0.2, 0.8 |
| Intake>85^th^ p* | 0 | 0, 0.1 | 0.1 | 0, 0.4 | 0 | 0, 0.2 | 0.1 | 0, 0.5 |
| **Nuts and Seeds** |  |  |  |  |  |  |  |  |
| No intake | 73.6 | 71.8, 75.2 | 72.5 | 69.6, 75.3 | 75.3 | 72, 78.3 | 69 | 66.9, 71.1 |
| Intake<recommendation | 25.8 | 24.2, 27.5 | 26.5 | 23.8, 29.5 | 23.5 | 20.6, 26.7 | 30.2 | 28.2, 32.4 |
| Intake=recommendation | 0.6 | 0.4, 0.8 | 0.9 | 0.4, 2 | 1.2 | 0.6, 2.1 | 0.7 | 0.4, 1.1 |
| Intake>recommendation | 0 | 0, 0 | 0 | 0, 0 | 0 | 0, 0 | 0 | 0, 0.2 |
| Intake>85^th^ p* | 0 | 0, 0.1 | 0 | 0, 0 | 0 | 0, 0.1 | 0.1 | 0, 0.5 |
| **Unsaturated oils** |  |  |  |  |  |  |  |  |
| No intake | 0.1 | 0, 0.2 | 0.1 | 0, 0.4 | 0 | 0, 0.1 | 0.1 | 0, 0.5 |
| Intake<recommendation | 97.3 | 96.7, 97.9 | 97.9 | 96.9, 98.6 | 95.3 | 93, 96.9 | 96 | 94.8, 96.9 |
| Intake=recommendation | 2.6 | 2, 3.2 | 2.1 | 1.4, 3 | 4.6 | 3.1, 7 | 3.9 | 3, 5.1 |
| Intake>recommendation | 0 | 0, 0.1 | 0 | 0, 0 |  |  | 0 | 0, 0 |
| Intake>85^th^ p* | 0 | 0, 0.1 | 0 | 0, 0 |  |  | 0 | 0, 0 |
| **Tubers** |  |  |  |  |  |  |  |  |
| No intake | 20.9 | 19.5, 22.4 | 22.4 | 20.1, 25 | 16.4 | 14, 19.2 | 13.8 | 12.4, 15.4 |
| Intake<recommendation | 64.1 | 62.3, 65.8 | 59.2 | 56.1, 62.4 | 68.3 | 64.7, 71.6 | 70 | 68, 72 |
| Intake=recommendation | 14.5 | 13.2, 15.8 | 17.6 | 15.3, 20 | 14.3 | 11.8, 17.2 | 15.3 | 13.7, 17.1 |
| Intake>recommendation | 0.6 | 0.4, 0.9 | 0.8 | 0.4, 1.5 | 1 | 0.6, 1.7 | 0.8 | 0.5, 1.4 |
| **Dairy** |  |  |  |  |  |  |  |  |
| No intake | 1.6 | 1.2, 2.1 | 1 | 0.5, 1.9 | 1 | 0.5, 1.9 | 0.7 | 0.5, 1 |
| Intake<recommendation | 30.7 | 28.9, 32.6 | 24.7 | 22.4, 27.1 | 32.1 | 28.9, 35.4 | 32.5 | 30.4, 34.7 |
| Intake=recommendation | 42.8 | 41.1, 44.4 | 50.1 | 46.8, 53.4 | 45.5 | 42.5, 48.5 | 50.7 | 48.5, 53 |
| Intake>recommendation | 24.9 | 23.2, 26.7 | 24.2 | 21.4, 27.2 | 21.5 | 18.6, 24.6 | 16 | 14.5, 17.7 |
| **Chicken/Poultry** |  |  |  |  |  |  |  |  |
| No intake | 10.9 | 9.9, 12 | 16.6 | 14.6, 18.9 | 11 | 9, 13.5 | 6.3 | 5.4, 7.3 |
| Intake<recommendation | 23.3 | 21.8, 24.8 | 35.5 | 32.3, 38.8 | 31.1 | 28.1, 34.2 | 25.4 | 23.5, 27.5 |
| Intake=recommendation | 50.8 | 49, 52.6 | 34.3 | 31.3, 37.5 | 41.6 | 38.1, 45.1 | 44.7 | 42.5, 46.9 |
| Intake>recommendation | 15 | 13.6, 16.5 | 13.5 | 11.5, 15.8 | 16.3 | 13.6, 19.5 | 23.6 | 21.6, 25.7 |
| **Egg** |  |  |  |  |  |  |  |  |
| No intake | 4.7 | 4, 5.5 | 3.9 | 3, 4.9 | 2.7 | 2, 3.7 | 2.6 | 2.1, 3.4 |
| Intake<recommendation | 20.7 | 19.3, 22.1 | 22.7 | 20, 25.6 | 20.1 | 17.5, 23.1 | 19.7 | 18, 21.6 |
| Intake=recommendation | 21.2 | 19.6, 23 | 21.4 | 18.9, 24.1 | 24.7 | 21.8, 27.9 | 22.2 | 20.4, 24.1 |
| Intake>recommendation | 53.4 | 51.6, 55.2 | 52.1 | 48.9, 55.2 | 52.4 | 48.9, 55.9 | 55.5 | 53.1, 57.8 |
| **Fish and Shellfish** |  |  |  |  |  |  |  |  |
| No intake | 21.2 | 19.8, 22.7 | 19.7 | 17.4, 22.2 | 19.7 | 16.8, 22.9 | 12.7 | 11.4, 14.2 |
| Intake<recommendation | 60.6 | 58.8, 62.4 | 62.8 | 59.6, 66 | 62.8 | 58.8, 66.6 | 69.5 | 67.2, 71.7 |
| Intake=recommendation | 17.5 | 16.1, 19.1 | 16.9 | 14.5, 19.6 | 15.6 | 13, 18.7 | 16.9 | 15.2, 18.8 |
| Intake>recommendation | 0.6 | 0.4, 0.9 | 0.5 | 0.3, 1.1 | 2 | 1, 3.7 | 0.8 | 0.5, 1.2 |
| **Red Meat** |  |  |  |  |  |  |  |  |
| No intake=recommendation | 0 |  | 0 |  | 0 |  | 0 |  |
| Intake<MTI^†^ | 44 | 42.2, 45.8 | 41.3 | 38.2, 44.4 | 49.9 | 46.3, 53.5 | 37.9 | 35.7, 40.2 |
| Intake>MTI^†^ | 56 | 54.2, 57.8 | 58.7 | 55.6, 61.8 | 50.1 | 46.5, 53.7 | 62.1 | 59.8, 64.3 |
| **Saturated Fats** |  |  |  |  |  |  |  |  |
| No intake=recommendation | 0 |  | 0 |  | 0 |  | 0 |  |
| Intake<MTI^†^ | 85.4 | 84, 86.7 | 81.1 | 78.3, 83.6 | 81.5 | 78.8, 83.9 | 80.7 | 78.8, 82.4 |
| Intake>MTI^†^ | 14.6 | 13.3, 16 | 18.9 | 16.4, 21.7 | 18.5 | 16.1, 21.2 | 19.3 | 17.6, 21.2 |
| **Added Sugars** |  |  |  |  |  |  |  |  |
| No intake=recommendation | 0 |  | 0 |  | 0 |  | 0 |  |
| Intake<MTI^†^ | 2.3 | 1.8, 2.9 | 2.2 | 1.5, 3.1 | 2.3 | 1.5, 3.6 | 1.9 | 1.4, 2.6 |
| Intake>MTI^†^ | 97.7 | 97.1, 98.2 | 97.8 | 96.9, 98.5 | 97.7 | 96.4, 98.5 | 98.1 | 97.4, 98.6 |

*85^th^ percentile between upper limit of intake recommendation and maximum intake in sample

†Maximum tolerable intake

**Supplementary Table 4. Distribution of the Mexican adolescent population according to component intake recommendation of the Global Burden of Disease Index, by survey year**

| **Intake vs. Recommendation Category** | 2006 | | 2012 | | 2016 | | 2018 | |
| --- | --- | --- | --- | --- | --- | --- | --- | --- |
|  | % | CI95% | % | CI95% | % | CI95% | % | CI95% |
| **Fruits** |  |  |  |  |  |  |  |  |
| No intake | 12.2 | 11.1, 13.4 | 8.7 | 7.1, 10.8 | 5.1 | 3.7, 6.9 | 8.9 | 7.8, 10.2 |
| Intake<recommendation | 70.0 | 68.3, 71.6 | 66.9 | 63.8, 69.8 | 70.2 | 67.1, 73.1 | 76.3 | 74.4, 78.2 |
| Intake≥recommendation | 17.8 | 16.3, 19.4 | 24.4 | 21.8, 27.2 | 24.7 | 22, 27.6 | 14.7 | 13.2, 16.4 |
| **Vegetables** |  |  |  |  |  |  |  |  |
| No intake | 0.1 | 0.1, 0.2 | 0 | 0, 0 | 0 | 0, 0 | 0.1 | 0, 0.2 |
| Intake<recommendation | 98.6 | 98, 99 | 96.2 | 94.7, 97.3 | 92.5 | 90.1, 94.3 | 96.6 | 95.6, 97.4 |
| Intake≥recommendation | 1.3 | 0.9, 1.9 | 3.8 | 2.7, 5.3 | 7.5 | 5.7, 9.8 | 3.3 | 2.6, 4.3 |
| **Legumes** |  |  |  |  |  |  |  |  |
| No intake | 4.2 | 3.5, 5.2 | 4.2 | 3.1, 5.6 | 3.2 | 2.2, 4.6 | 2.8 | 2.1, 3.7 |
| Intake<recommendation | 95.7 | 94.7, 96.5 | 94.3 | 92.7, 95.5 | 94.2 | 92.5, 95.6 | 95.9 | 94.9, 96.7 |
| Intake≥recommendation | 0.1 | 0, 0.2 | 1.6 | 1, 2.4 | 2.6 | 1.7, 3.9 | 1.3 | 0.9, 1.9 |
| **High-Fiber Cereals** |  |  |  |  |  |  |  |  |
| No intake | 1.7 | 1.4, 2.2 | 2.8 | 2, 3.9 | 1.0 | 0.5, 1.8 | 1.0 | 0.6, 1.5 |
| Intake<recommendation | 38.0 | 35.7, 40.2 | 41.8 | 38.7, 45 | 28.8 | 25.7, 32 | 36.3 | 34.1, 38.5 |
| Intake≥recommendation | 60.3 | 58, 62.6 | 55.3 | 52.1, 58.5 | 70.3 | 67, 73.4 | 62.7 | 60.5, 64.9 |
| **Nuts and Seeds** |  |  |  |  |  |  |  |  |
| No intake | 73.6 | 71.8, 75.2 | 72.5 | 69.6, 75.3 | 75.3 | 72, 78.3 | 69 | 66.9, 71.1 |
| Intake<recommendation | 25.3 | 23.6, 27 | 25.1 | 22.5, 27.9 | 22.8 | 19.9, 26 | 29.5 | 27.5, 31.7 |
| Intake≥recommendation | 1.2 | 0.9, 1.5 | 2.4 | 1.3, 4.1 | 1.9 | 1.1, 3.3 | 1.4 | 1, 2 |
| **Milk** |  |  |  |  |  |  |  |  |
| No intake | 19.4 | 18, 20.8 | 20 | 17.5, 22.6 | 16.8 | 14.3, 19.7 | 13.9 | 12.4, 15.6 |
| Intake<recommendation | 74.1 | 72.5, 75.6 | 71.4 | 68.2, 74.3 | 74.0 | 70.9, 76.9 | 82.0 | 80.2, 83.6 |
| Intake≥recommendation | 6.6 | 5.7, 7.6 | 8.7 | 6.8, 11 | 9.2 | 7.2, 11.6 | 4.1 | 3.5, 4.9 |
| **Fiber** |  |  |  |  |  |  |  |  |
| No intake | 0 | - | 0 | - | 0 | - | 0 | - |
| Intake<recommendation | 68 | 66.1, 69.8 | 59.9 | 56.8, 62.9 | 52.1 | 48.6, 55.6 | 61.6 | 59.3, 63.9 |
| Intake≥recommendation | 32 | 30.2, 33.9 | 40.1 | 37.1, 43.2 | 47.9 | 44.4, 51.4 | 38.4 | 36.1, 40.7 |
| **Calcium** |  |  |  |  |  |  |  |  |
| No intake | 0 | - | 0 | - | 0 | - | 0 | - |
| Intake<recommendation | 84.3 | 82.5, 86 | 76.5 | 73.7, 79.1 | 71.2 | 68, 74.1 | 79.7 | 77.8, 81.6 |
| Intake≥recommendation | 15.7 | 14, 17.5 | 23.5 | 20.9, 26.3 | 28.8 | 25.9, 32 | 20.3 | 18.4, 22.2 |
| **Omega-3 Fatty Acids** |  |  |  |  |  |  |  |  |
| No intake |  |  |  |  |  |  |  |  |
| Intake<recommendation | 92.6 | 91.5, 93.5 | 93.9 | 92.2, 95.2 | 89.5 | 86.8, 91.8 | 90.8 | 89.3, 92 |
| Intake≥recommendation | 7.4 | 6.5, 8.5 | 6.1 | 4.8, 7.8 | 10.5 | 8.2, 13.2 | 9.2 | 8, 10.7 |
| **Omega-6 Fatty Acids** |  |  |  |  |  |  |  |  |
| No intake |  | - | 0 | - | 0 | - | 0 | - |
| Intake<recommendation | 100 | 99.9, 100 | 100 | 0, 0 | 100 | 99.9, 100 | 99.9 | 99.7, 100 |
| Intake≥recommendation | 0 | 0, 0.1 | 0 | 0, 0 | 0 | 0, 0.1 | 0.1 | 0, 0.3 |
| **Red Meat** |  |  |  |  |  |  |  |  |
| No intake | 10.2 | 9.1, 11.3 | 7.2 | 5.9, 8.9 | 8.6 | 7, 10.6 | 5.7 | 4.8, 6.8 |
| Intake<recommendation | 34.9 | 33.2, 36.5 | 30.9 | 28, 33.9 | 35.7 | 32.3, 39.3 | 30.6 | 28.6, 32.6 |
| Intake=recommendation | 11.3 | 10.1, 12.6 | 10.8 | 8.9, 13.1 | 13.1 | 11, 15.4 | 13.1 | 11.7, 14.6 |
| Intake>recommendation | 43.7 | 41.7, 45.7 | 51.0 | 47.8, 54.3 | 42.6 | 39.2, 46.1 | 50.6 | 48.4, 52.9 |
| **Processed Meats** |  |  |  |  |  |  |  |  |
| No intake=recommendation | 0 | - | 0 | - | 0 | - | 0 | - |
| Intake<MTI^†^ | 33.0 | 31, 35 | 20.9 | 18.5, 23.4 | 23.3 | 20.4, 26.5 | 19.0 | 17.2, 21.0 |
| Intake>MTI^†^ | 67.0 | 65, 69 | 79.1 | 76.6, 81.5 | 76.7 | 73.5, 79.6 | 81.0 | 79.0, 82.8 |
| **Sugar-Sweetened Beverages** |  |  |  |  |  |  |  |  |
| No intake=recommendation | 0 | - | 0 | - | 0 | - | 0 | - |
| Intake<MTI^†^ | 8.4 | 7.5, 9.5 | 7.0 | 5.4, 9.1 | 4.3 | 3.1, 6 | 3.2 | 2.5, 4.1 |
| Intake>MTI^†^ | 91.6 | 90.5, 92.5 | 93 | 90.9, 94.6 | 95.7 | 94, 96.9 | 96.8 | 95.9, 97.5 |
| **Trans Fatty Acids** |  |  |  |  |  |  |  |  |
| No intake=recommendation | 0 | - | 0 | - | 0 | - | 0 | - |
| Intake<MTI^†^ | 59.9 | 57.8, 62 | 51.7 | 48.4, 54.9 | 59.2 | 56.1, 62.2 | 60.6 | 58.2, 62.9 |
| Intake>MTI^†^ | 40.1 | 38, 42.2 | 48.3 | 45.1, 51.6 | 40.8 | 37.8, 43.9 | 39.4 | 37.1, 41.8 |
| **Sodium** |  |  |  |  |  |  |  |  |
| No intake=recommendation | 0 | - | 0 | - | 0 | - | 0 | - |
| Intake<MTI^†^ | 83.3 | 81.8, 84.8 | 71.2 | 68.1, 74.1 | 76.1 | 73, 78.8 | 79.7 | 77.7, 81.5 |
| Intake>MTI^†^ | 16.7 | 15.2, 18.2 | 28.8 | 25.9, 31.9 | 23.9 | 21.2, 27 | 20.3 | 18.5, 22.3 |

†Maximum tolerable intake

**Supplementary Table 5. Distribution of the Mexican adolescent population according to component intake recommendation of the Mexican Dietary Guidelines Index, by survey year**

| **Intake vs. Recommendation Category** | 2006 | | 2012 | | 2016 | | 2018 | |
| --- | --- | --- | --- | --- | --- | --- | --- | --- |
|  | % | CI95% | % | CI95% | % | CI95% | % | CI95% |
| **Vegetables** |  |  |  |  |  |  |  |  |
| No intake | 0.1 | 0, 0.2 | 0 | 0, 0 | 0 | 0, 0 | 0 | 0, 0.2 |
| Intake<recommendation | 94.2 | 93.2, 95.1 | 92.5 | 90.4, 94.2 | 90.8 | 88.6, 92.6 | 94.2 | 92.8, 95.3 |
| Intake≥recommendation | 5.7 | 4.8, 6.7 | 7.5 | 5.8, 9.6 | 9.2 | 7.3, 11.4 | 5.8 | 4.7, 7.1 |
| **Fruits** |  |  |  |  |  |  |  |  |
| No intake | 7.8 | 6.8, 8.9 | 6.7 | 5.2, 8.6 | 3.6 | 2.5, 5.3 | 4.5 | 3.7, 5.5 |
| Intake<recommendation | 75.7 | 74, 77.3 | 69.7 | 66.6, 72.7 | 75.6 | 72.9, 78.2 | 78 | 76.1, 79.8 |
| Intake≥recommendation | 16.5 | 15.1, 18.1 | 23.5 | 20.8, 26.5 | 20.7 | 18.3, 23.3 | 17.5 | 15.8, 19.3 |
| **Cereals** |  |  |  |  |  |  |  |  |
| No intake | 0 | - | 0 | - | 0 | -- | 0 |  |
| Intake<recommendation | 28.8 | 26.9, 30.8 | 37.7 | 34.6, 40.8 | 32.8 | 29.5, 36.4 | 41.4 | 39.1, 43.7 |
| Intake≥recommendation | 71.2 | 69.2, 73.1 | 62.3 | 59.2, 65.4 | 67.2 | 63.6, 70.5 | 58.6 | 56.3, 60.9 |
| **Legumes** |  |  |  |  |  |  |  |  |
| No intake | 3.8 | 3.1, 4.8 | 4 | 2.9, 5.4 | 2.9 | 1.9, 4.3 | 2.4 | 1.8, 3.3 |
| Intake<recommendation | 96.2 | 95.2, 96.9 | 96 | 94.6, 97.1 | 97.1 | 95.7, 98.1 | 97.5 | 96.7, 98.1 |
| Intake≥recommendation | 0 | - | 0 | - | 0 | - | 0 | 0, 0.2 |
| **Low-Fat Dairy** |  |  |  |  |  |  |  |  |
| No intake | 10.7 | 9.6, 11.9 | 13.2 | 11.3, 15.4 | 14.2 | 11.8, 17.1 | 8.5 | 7.3, 9.9 |
| Intake<recommendation | 74.9 | 73.2, 76.6 | 74.1 | 71.3, 76.8 | 72.7 | 69.3, 75.9 | 81 | 79.1, 82.7 |
| Intake≥recommendation | 14.4 | 12.8, 16.2 | 12.6 | 10.6, 15 | 13 | 10.7, 15.8 | 10.5 | 9.2, 12 |
| **Low-Fat Animal-Based Foods** |  |  |  |  |  |  |  |  |
| No intake | 3.8 | 3.1, 4.6 | 2.9 | 2.1, 4.1 | 1.9 | 1.2, 3.2 | 2.1 | 1.5, 2.8 |
| Intake<recommendation | 88.4 | 87.3, 89.5 | 90.3 | 88.1, 92.1 | 87.4 | 84.9, 89.6 | 83 | 81.1, 84.8 |
| Intake≥recommendation | 7.8 | 6.9, 8.8 | 6.8 | 5.3, 8.8 | 10.6 | 8.6, 13 | 14.9 | 13.2, 16.8 |
| **Plain Water** |  |  |  |  |  |  |  |  |
| No intake | 6 | 5, 7.1 | 12.7 | 10.4, 15.5 | 3 | 2.3, 4.1 | 3.8 | 3, 4.8 |
| Intake<recommendation | 82.1 | 80.6, 83.5 | 72.8 | 69.5, 75.8 | 76.1 | 73.3, 78.7 | 75.3 | 73.3, 77.2 |
| Intake≥recommendation | 11.9 | 10.8, 13.1 | 14.5 | 12.4, 16.9 | 20.8 | 18.4, 23.6 | 20.9 | 19.2, 22.8 |
| **High-Fat Animal-Based Foods** |  |  |  |  |  |  |  |  |
| No intake=recommendation | 0 | - | 0 | - | 0 | - | 0 | - |
| Intake<MTI^†^ | 49.4 | 47.5, 51.4 | 41.5 | 38.4, 44.7 | 51.8 | 47.9, 55.6 | 41.7 | 39.6, 43.9 |
| Intake>MTI^†^ | 50.6 | 48.6, 52.5 | 58.5 | 55.3, 61.6 | 48.2 | 44.4, 52.1 | 58.3 | 56.1, 60.4 |
| **Added Sugars** |  |  |  |  |  |  |  |  |
| No intake=recommendation | 0 | - | 0 | - | 0 | - | 0 | - |
| Intake<MTI^†^ | 18.5 | 17.1, 20.1 | 11.9 | 9.8, 14.4 | 18 | 15.7, 20.5 | 12.4 | 11, 14 |
| Intake>MTI^†^ | 81.5 | 79.9, 82.9 | 88.1 | 85.6, 90.2 | 82 | 79.5, 84.3 | 87.6 | 86, 89 |
| **Foods rich in fats** |  |  |  |  |  |  |  |  |
| No intake=recommendation | 0 | - | 0 | - | 0 | - | 0 | - |
| Intake<MTI^†^ | 75.8 | 74.1, 77.4 | 71.3 | 68.3, 74.2 | 72.2 | 69.2, 75.1 | 61.7 | 59.4, 63.9 |
| Intake>MTI^†^ | 24.2 | 22.6, 25.9 | 28.7 | 25.8, 31.7 | 27.8 | 24.9, 30.8 | 38.3 | 36.1, 40.6 |

†Maximum tolerable intake
